# Characterization of Long Non-Coding RNA Profiles in Porcine Granulosa Cells of Healthy and Atretic Antral Follicles: Implications for a Potential Role in Apoptosis

**DOI:** 10.3390/ijms22052677

**Published:** 2021-03-06

**Authors:** Li Meng, Kun Zhao, Chi Chiu Wang, Jian Tao, Zhenfang Wu, Katja Teerds, Shouquan Zhang

**Affiliations:** 1 National Engineering Research Center of Breeding Swine Industry, College of Animal Science, South China Agricultural University, Guangzhou 510642, China; limeng@scau.edu.cn (L.M.); zhao971111@gmail.com (K.Z.); taojian19922020@gmail.com (J.T.); wzfemail@163.com (Z.W.); 2 Guangdong Provincial Key Lab of Agro-Animal Genomics and Molecular Breeding, Key Lab of Chicken Genetics, Breeding and Reproduction, Ministry of Agriculture, South China Agricultural University, Guangzhou 510642, China; 3 Department of Obstetrics & Gynaecology, Li Ka Shing Institute of Health Sciences, School of Biomedical Sciences, The Chinese University of Hong Kong, Shatin, Hong Kong, China; ccwang@cuhk.edu.hk; 4 Human and Animal Physiology, Wageningen University, De Elst 1, 6708 WD Wageningen, The Netherlands

**Keywords:** lncRNAs, transcriptome profiles, antral follicular atresia

## Abstract

Long non-coding RNAs (lncRNAs) play important roles in multiple biological processes including ovarian follicular development. Here we aimed to gain novel information regarding lncRNAs transcriptome profiles in porcine granulosa cells of advanced atretic antral (AA) and healthy antral (HA) follicles using RNA-seq. A total of 11,321 lncRNAs including 10,813 novel and 508 annotated lncRNAs were identified, of which 173 lncRNAs were differentially expressed (DE-lncRNAs); ten of these were confirmed by qRT-PCR. Gene Ontology indicated that DE-lncRNAs associated with developmental processes were highly enriched. Pathway analysis demonstrated predicted cis- and trans-targets of DE-lncRNAs. Potential mRNA targets of up-regulated DE-lncRNAs were mainly enriched in apoptosis related pathways, while targeted genes of downregulated DE-lncRNAs were primarily enriched in metabolism and ovarian steroidogenesis pathways. Linear regression analyses showed that expression of upregulated DE-lncRNAs was significantly associated with apoptosis related genes. NOVEL_00001850 is the most-downregulated DE-lncRNA (FDR = 0.04, FC = −6.53), of which miRNA binding sites were predicted. KEGG analysis of its downregulated target genes revealed that ovarian steroidogenesis was the second most highlighted pathway. qRT-PCR and linear regression analysis confirmed the expression and correlation of its potential targeted gene, *CYP19A1*, a key gene involved in estradiol synthesis. Our results indicate that lncRNAs may participate in granulosa cells apoptosis and thus antral follicular atresia.

## 1. Introduction

The main function of the ovary is the production of steroid hormones necessary for the development of female secondary sex characteristics, and to provide an optimal environment for oocyte maturation and release [1]. Ovarian follicular development is regulated by various factors including gonadotropins and growth factors all involved in the process of selecting the best follicles to ovulate [2]. The vast majority of follicles however fail to reach the preovulatory stage, and instead undergo degeneration by a process named atresia [1]. Although atresia can be considered a physiologically normal process, which ensures that only oocytes from the best quality follicles will ovulate, dysregulated atresia can lead to reproductive diseases such as polycystic ovary syndrome (PCOS) and premature menopause in women at reproductive age [2]. 

Non-protein-coding transcripts have been detected by means of next-generation sequencing, a technique with unprecedented resolution [3]. It has come as quite a surprise that less than 2% of the human genome encodes proteins; the majority of nucleotides are not transcribed into proteins, representing noncoding transcripts [4]. Among the various types of noncoding transcripts, such as miRNAs and circular RNAs (circRNAs), a class referred to as long noncoding RNAs (lncRNAs) is attracting increasing attention. lncRNAs are defined as transcripts of more than 200 nucleotides consisting of a diverse class of intergenic transcripts (between protein-coding genes), enhancer RNAs (eRNAs), and sense or antisense transcripts that overlap other genes [4]. Accumulating evidence shows that lncRNAs are not only involved in many physiological processes including development, differentiation, cell proliferation, and apoptosis, but also play a role in pathological conditions such as diabetes and tumorigenesis [3]. lncRNAs have been proposed to execute diverse functions, including transcriptional regulation acting in cis, influencing the expression of nearby genes, or leaving the site of transcription and carrying out an array of roles in trans [5]. These trans lncRNAs can be divided into at least two groups: a group of lncRNAs that regulate gene expression at regions distant from their transcription site and a group of lncRNAs that regulate RNA molecules such as miRNAs through a mechanism termed competing endogenous RNAs (ceRNAs) [3]. 

lncRNAs have been associated with ovarian follicular development, although the number of studies is limited. Ovarian lncRNAs were first identified by RNA sequencing in human cumulus granulosa cells. This study showed the presence of 89 differentially expressed lncRNAs between compact cumulus granulosa cells and expanded cumulus cells, suggesting a role for lncRNAs in cumulus expansion [6]. In line with this, Xu et al. identified over 600 differentially expressed lncRNAs in mice cumulus granulosa cells isolated from cumulus oocyte complexes (COCs) that produced either good- or poor-quality embryos, indicating that lncRNAs may indirectly affect oocyte quality [7]. lncRNAs have also been identified in mural granulosa cells. For example, lncRNA-Amhr2, is transcribed upstream of the mouse AMH type II receptor (Amhr2) gene. Knockdown of lncRNA-Amhr2 results in a decrease in Amhr2 messenger at RNA level, while a transient reporter gene assay shows that lncRNA-Amhr2 activation increases Amhr2 promoter activity. These results provide evidence that lncRNA-Amhr2 may play a role in Amhr2 gene activation in ovarian mural granulosa cells by enhancing promoter activity [8]. 

Besides being involved in normal ovarian follicular development, aberrant expression of lncRNAs has been reported in pathological ovarian conditions such as PCOS. Huang et al. observed a difference in lncRNA expression pattern between cumulus cells from women with PCOS and normal healthy women [9]. In line with this observation, Jiao et al. reported that the lncRNA expression profile in follicular fluid of women with PCOS differed from normal healthy women [10].

Unlike miRNAs which have been extensively studied, the involvement of lncRNA in antral follicular atresia is less well unknown. The aim of the present study is therefore to unravel the role of lncRNAs in the process of antral follicular atresia using next generation RNA sequencing. The well-defined start of the follicular phase and easy accessibility to large quantities of ovarian tissues makes the pig a very suitable animal model to investigate ovarian follicular physiology and thus we have chosen to use this model in our investigation. We postulate that granulosa cell apoptosis in atretic porcine follicles will coincide with differential expression of lncRNAs involved in pathways related to follicular survival such as steroid synthesis and metabolism.

## 2. Results

### 2.1. RNA Sequencing Identifies Specific lncRNAs in Granulosa Cells of Healthy and Atretic Porcine Antral Follicles

**Figure 1:**
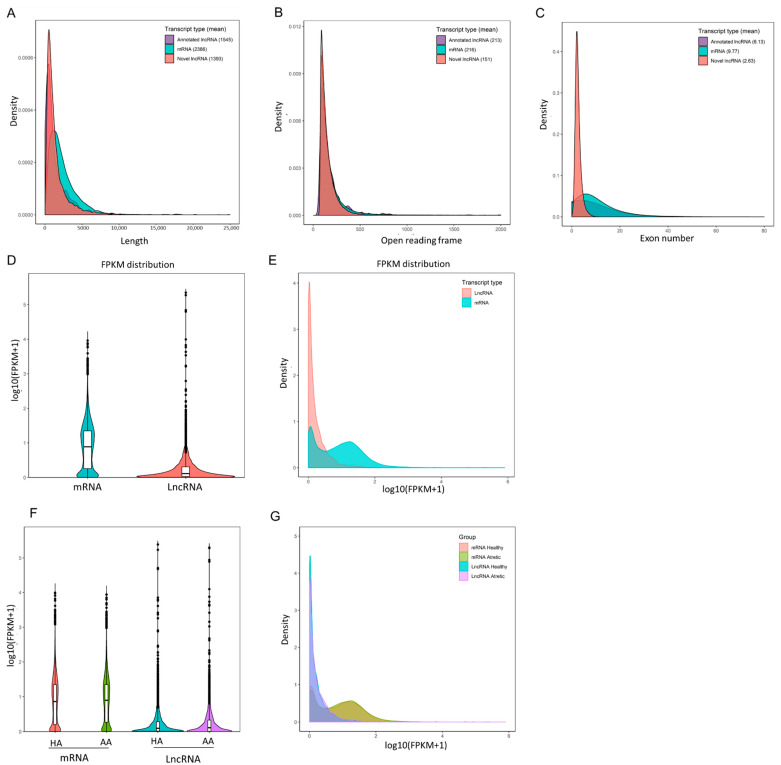
Characterization of long non-coding RNAs (lncRNAs) and messenger RNAs (mRNAs) from antral follicle granulosa cells. (**A**–**C**), the transcript length, open reading frames, and exon numbers of annotated lncRNAs, novel lncRNAs and mRNAs. (**D**,**E**) Violin plot and density distribution diagram showing the expression features of lncRNAs and mRNAs in granulosa cells from porcine antral follicles. (**F**,**G**) Violin plot and density distribution diagram showing the expression features of lncRNAs and mRNAs in granulosa cells from healthy antral (HA) and atretic antral (AA) follicles, respectively. FPKM, fragments per kilobase million.

RNA sequencing data were analyzed from six different pooled porcine antral follicle samples (for details see Materials and Methods). After filtering out low-quality reads and removing adaptor sequences, around 640.6 million reads (paired end ~96.1 bp in length) were obtained. Approximately 86.68% of the total clean reads mapped to the *S. scrofa* genome assembly (version 10.2); in total 11,321 lncRNAs including 10,813 novel and 508 annotated lncRNA transcripts were assembled. 96.18% of the assembled lncRNAs consisted of predicted novel lncRNAs, 2.32% of processed transcripts, 0.96% of miscRNAs, 0.44% of lincRNAs and 0.10% of antisense RNAs. In agreement with a previous study [10], our data showed that the identified porcine granulosa cell lncRNAs had a shorter total length than coding mRNA transcripts (Figure 1A) as well as reduced length of the open reading frame (Figure 1B), and possessed fewer exons (Figure 1C). The mRNA content was significantly higher than the lncRNAs content in antral granulosa cells samples (Figure 1D,E), but there was no significant difference between transcript levels in granulosa cells from healthy and atretic antral follicles (Figure 1F,G). 

### 2.2. Differences in Gene Expression Patterns of lncRNAs in Granulosa Cells of Healthy and Atretic Antral Follicles

**Figure 2:**
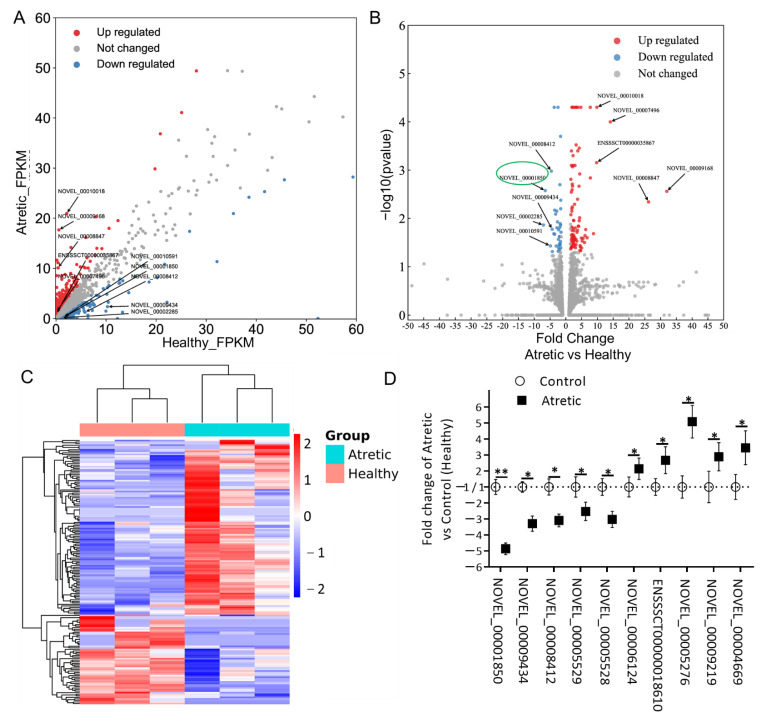
Expression profiles of differentially expressed lncRNAs (DE-lncs). (**A**) Scatter plot assessing the overall distribution of DE-lncs between HA and AA follicles. Values on the horizontal and vertical axis represent the averaged normalized signal values of each group (log2 transformed). The red and blue dots indicate lncRNAs with fold change (FC) ≥ 1.5. Top 5 up and downregulated transcripts were labeled. (**B**) Volcano plot visualizing the statistical difference of the DE-lncs. The horizontal axis represents the FC of detected transcripts and the vertical axis represents the *p*-value. The red dots in the plot denote the upregulated transcripts (FC ≥ 1.5 and *p* ≤ 0.05, respectively); the blue dots represent the downregulated transcripts (FC ≤ −1.5 and *p* ≤ 0.05, respectively). Top 5 up and downregulated transcripts were labeled. (**C**) Hierarchical clustering showing the expression profiles of all the DE-lncs. Rows represent DE-lncs while columns represent different samples. The DE-lncs were classified according to Pearson correlation. (**D**) Relative lncRNA expression measured by qRT-PCR. lncRNA expression is calculated as fold change of AA (*n* = 3) over HA (*n* = 3) follicles. lncRNA expression in HA was set to 1 for up-regulated genes and -1 for down-regulated genes. *, *p* < 0.05; **, *p* < 0.01; HA, healthy antral follicles; AA, atretic antral follicles.

To further identify potential lncRNA transcripts involved in the process of antral follicular atresia, the expression patterns of lncRNAs were analyzed. Differentially expressed (DE) lncRNAs were identified using scatter and volcano plot filtering (Figure 2A,B). A total of 173 lncRNA transcripts were differentially expressed in granulosa cells from AA follicles compared with HA follicles, with 114 transcripts being upregulated and 59 downregulated. All up-regulated and down-regulated lncRNA transcripts were further evaluated by unsupervised hierarchical clustering analysis (Figure 2C), which showed a good correlation within groups and a clear distinction between HA and AA follicles.

Among the DE-lncRNA transcripts, NOVEL_00009168 (FC = 31.98) was the most significantly upregulated lncRNA, whereas NOVEL_00002285 (FC = −7.07) was the most significantly downregulated lncRNA (Table 1).

### 2.3. qRT-PCR Validation of lncRNAs

To validate the results of the RNA-seq data, we analyzed ten DE-lncRNAs by qRT-PCR in granulosa cells from atretic antral (AA) and healthy antral (HA) follicles, respectively. These lncRNA transcripts were chosen based on their length (≤3500 nucleotides), fragments per kilobase million (FPKM) (≥1.0), and FC (≥2.0). The selected and by qRT-PCR analyzed DE-lncRNAs included five down-regulated (NOVEL_00001850, NOVEL_00009434, NOVEL_00008412, NOVEL_00005529, and NOVEL_00005528) and five up-regulated (NOVEL_00006124, ENSSSCT00000018610, NOVEL_00005276, NOVEL_00009219, and NOVEL_00004669) transcripts (Figure 2D). 

### 2.4. Systematic Functional Analysis of Differentially Expressed lncRNAs in Granulosa Cells of HA and AA Follicles

The potential targets of all DE-lncRNAs in cis and trans-regulation were analyzed to predict the functions of these lncRNAs. For the cis- regulation of lncRNAs, we searched for differentially expressed mRNAs that were situated 50 kb upstream and downstream of the lncRNAs. RIsearch software was used to analyze the trans-regulated mRNAs applying a threshold of free energy < 100. As a result of this analysis, 27 cis- and 652 trans- differentially expressed mRNA transcripts for DE-lncRNAs were identified. 

**Figure 3:**
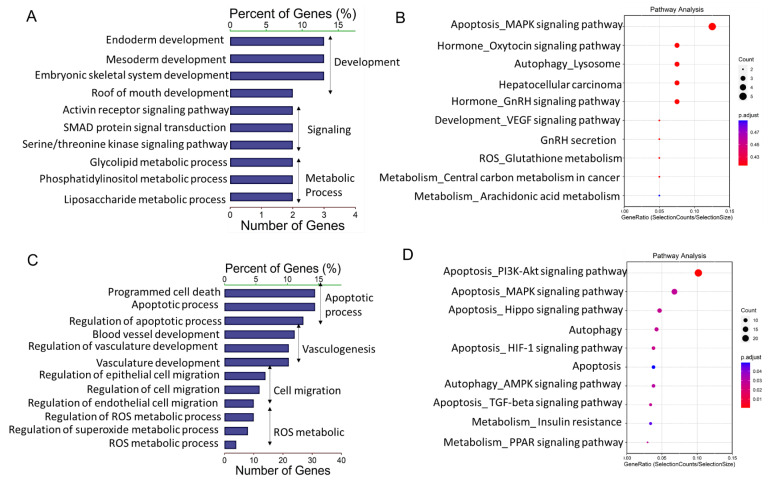
Gene Ontology and Kyoto Encyclopedia of Genes and Genomes (KEGG) analysis of differentially expressed lncRNAs (DE-lncs) target genes. (**A**,**C**) GO categories (biological process) of differential lncRNA cis-target genes (**A**) and trans-target genes (**C**) in granulosa cells from AA versus HA follicles. (**B**,**D**) KEGG analysis of differential lncRNA cis-target genes (**B**) and trans-target genes (**D**) in granulosa cells from HA versus AA follicles. The size and color of each bubble represents the number of genes in each pathway and P value respectively. HA, heathy antral; AA, atretic antral.

To elucidate the possible functional significance of the observed difference in lncRNA expression between granulosa cells from HA and AA follicles, GO and KEGG analysis were performed for the cis- and trans-targets. GO analysis showed that DE-lncRNAs cis-targets were significantly associated with various developmental and metabolic processes (Figure 3A). KEGG pathway analysis indicated that the apoptosis-related MAPK signaling pathway, oxytocin and GnRH related signaling pathways were highly enriched in the differentially expressed lncRNAs (Figure 3B).

GO analysis revealed further that a number of DE-lncRNAs trans-targets were significantly associated with developmental and cell migration processes (Figure 3C). In addition, pathway analysis of these potential targeted genes showed that reactive oxygen species (ROS)-related metabolic processes and apoptotic processes were also highly enriched (Figure 3C). In line with these results, KEGG pathway analysis made clear that the targeted transcripts were primarily linked to apoptosis-related pathways including “PI3K-Akt signaling pathway”, “MAPK signaling pathway”, “Hippo signaling pathway”, “HIF-1 signaling pathway”, and “Apoptosis”. Moreover, it is noteworthy that autophagy related pathways including “Autophagy” and “AMPK signaling pathway” were significantly enriched as well (Figure 3D).

### 2.5. Prediction of miRNA Binding Sites and lncRNA-miRNA-mRNA Network Analysis

**Figure 4:**
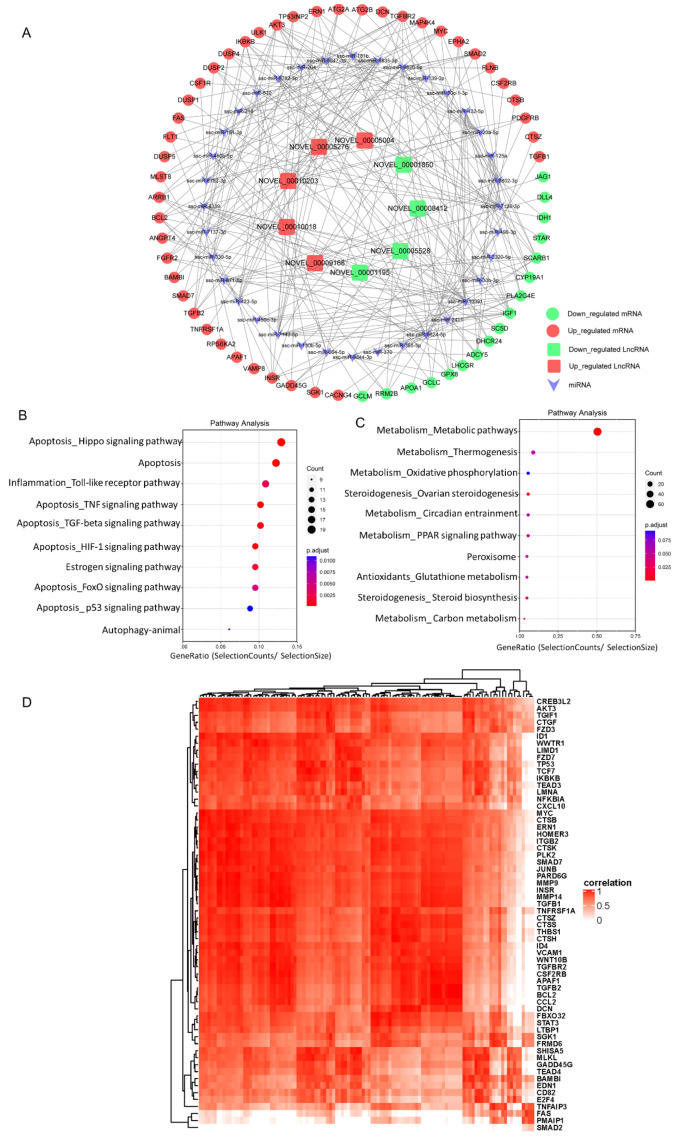
The lncRNA-miRNA-mRNA interaction network construction and Kyoto Encyclopedia of Genes and Genomes (KEGG) analysis of differentially expressed lncRNAs (DE-lncs) target genes via miRNAs. (**A**) Representative lncRNA-miRNA-mRNA interaction network. (**B**) KEGG analysis of potential up-regulated targets via miRNA of top10 upregulated lncRNAs in granulosa cells from AA follicles versus HA follicles. (**C**) KEGG analysis of potential down-regulated targets via miRNA of top10 downregulated lncRNAs in granulosa cells from AA follicles versus HA follicles. The size and color of each bubble represents the number of genes in each pathway and P value respectively. (**D**) A correlation heatmap between upregulated lncRNAs and apoptosis related mRNAs. Rows represent upregulated apoptosis related mRNAs and the columns represent upregulated lncRNAs. A red color indicates a positive correlation. HA, heathy antral; AA, atretic antral.

Based on our RNA-seq data a lncRNAs-miRNA-mRNA interaction network was constructed using Cytoscape. The top five up-regulated lncRNAs and the top five down-regulated lncRNAs were selected to identify lncRNA-miRNA interactions based on TargetScan and miRanda analysis (Figure 4A). KEGG pathway analysis of the targeted up-regulated mRNAs indicated that cell programming death pathways like autophagy and apoptosis-related pathways including “Hippo signaling pathway”, “Apoptosis”, “TNF signaling pathway”, “TGF-beta signaling pathway”, “HIF-1 signaling pathway”, “FoxO signaling pathway”, and “p53 signaling pathway” were highly enriched (Figure 4B). Downregulated potential mRNA targets were primarily associated with metabolic pathways including “Metabolic pathways”, “Thermogenesis”, “Oxidative phosphorylation”, “Circadian entrainment”, and “PPAR signaling pathway”. Additionally, ovarian steroidogenesis pathways including “Ovarian steroidogenesis” and “Steroid biosynthesis” were also enriched in the downregulated potential mRNA targets (Figure 4C). In line with these results, a correlation analysis based on Pearson coefficient showed that the upregulated lncRNAs were significantly associated with mRNA expression of 62 apoptosis related genes (Figure 4D), indicative of a direct role of lncRNAs in granulosa cells apoptosis.

### 2.6. Putative Role of DE-lncRNA NOVEL_00001850 in Antral Follicles

**Figure 5:**
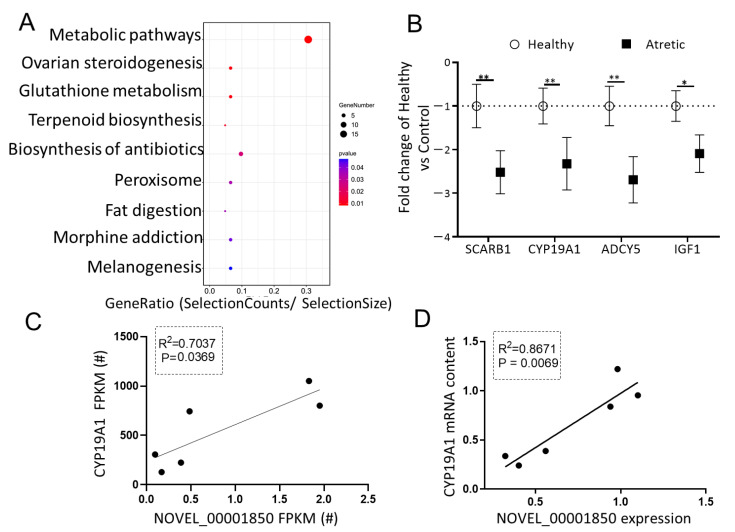
The putative role of NOVEL_00001850. (**A**) KEGG analysis of potential down-regulated targets of NOVEL_00001850 top three miRNA binding sites. The size and color of each bubble represents the number of genes in each pathway and P value respectively. (**B**) Validation of selected downregulated genes using qRT-PCR. Gene expression as fold change of atretic over healthy follicles (*n* = 3), with no change indicated as 1. Healthy: open circles, Atretic: filled squares. (**C**) Correlation of FPKM levels by RNA-Seq of NOVEL_00001850 and *CYP19A1*. (**D**) Correlation of expression levels by qRT-PCR of NOVEL_00001850 and *CYP19A1*. Data as mean ± SD. *, *p* < 0.05; **, *p* < 0.01.

To investigate the potential significance of DE-lncRNAs in antral follicular atresia, one of the most downregulated lncRNAs in GC of AA follicles, NOVEL_00001850 (FC = −6.520, FDR = 0.04) with miRNA binding sites, attracted our interest. The NOVEL_00001850-miRNA-mRNA network was constructed using Cytoscape to visualize interactions (Figure 4A). The top three sponged miRNAs (ssc-miR-125a, ssc-miR-1224, ssc-miR-1285) and corresponding putative mRNA targeted genes of these miRNAs were identified. Following KEGG pathway analysis of the downregulated potential targeted genes of NOVEL_00001850 (Figure 5A), we observed that next to metabolic pathways the top enriched processes referred to ovarian steroidogenesis, a process directly associated with follicular development and antral follicular atresia. In line with the RNA-seq data, the mRNA content of four genes related to ovarian steroidogenesis—*SCARB1, CYP19A1, IGF1* and *ADCY5*—was significantly down-regulated in granulosa cells of AA follicles compared to granulosa cells of HA follicles (Figure 5B). To determine whether the down-regulation of the expression of these four potential targeted mRNAs was directly associated with the expression of NOVEL_00001850, linear regression analyses were performed. Interestingly, these analyses showed that expression of NOVEL_00001850 was significantly associated with the mRNA content of *CYP19A1*, a key gene involved in estradiol synthesis suppressed by ssc-miR-125a (Figure 5C,D).

## 3. Discussion

In the current study, we employ RNA sequencing to identify lncRNA profiles in granulosa cells of antral follicles, and to further identify differences in expression profiles between HA and AA follicles using the porcine ovary as a model. Additionally, combining mRNA and lncRNA profiles, a multi-level analysis is performed to investigate how differential expression profiles of lncRNA and mRNA contribute to antral follicular atresia. In total 173 lncRNA transcripts were observed to be differently expressed in the granulosa cells of AA follicles compared with HA follicles. The RNA-seq data was further confirmed by qRT-PCR. KEGG analysis of potential target genes of the DE-lncRNAs showed that apoptosis related pathways were highly enriched. We further showed that NOVEL_00001850 was one of the most relevant down-regulated DE-lncRNAs with miRNA binding sites. It was confirmed that the potentially targeted gene of NOVEL_00001850 was *CYP19A1*, a gene involved in estradiol synthesis. Based on these results, we hypothesize that lncRNAs may potentially play a role in follicular atresia and granulosa cells apoptosis by negatively influencing estradiol synthesis.

Evolutionary conservation among mammalian species has long been a confusing characteristic of lncRNAs. Although recent studies have shown that expression and sequence of most identified lncRNAs do not seem to be conserved across mammals, it remains possible that some lncRNAs may have a conserved function. A recent study using comparative sequence, structural, and functional analyses has indeed provided evidence for evolutionary conservation of the human lncRNA *JPX* [11]. It was found that despite of a deep divergence of the nucleotide sequences and RNA secondary structures between human lncRNA *JPX* and its mouse homolog, lncRNA *Jpx,* both lncRNAs showed robust binding to CTCF, a protein that is crucial to *Jpx*’s role in X chromosome inactivation. Furthermore, an in vitro functional rescue experiment using *Jpx*-deleted mutant cells showed that human *JPX* can functionally complement the loss of *Jpx* in mouse embryonic stem cells. Many lncRNAs may also have “functional orthologs”: genes with similar function but no ancestral relationship [12]. Support for functional orthology comes from recently identified human lncRNA *XIST.* Although *XIST* is not found in marsupials, an opossum lncRNA called *RSX* was shown to have a similar function. While *RSX* is capable of silencing the X chromosome in mice, it shares no ancestral relationship with *XIST*, playing a role in the X chromosome inactivation. These findings support a model for functional conservation of lncRNAs independent from sequence and structural divergence, which is in line with our finding that porcine ovarian lncRNAs, similar with human and mouse, may play the functional role in regulation of granulosa cells apoptosis.

Investigations regarding the role of lncRNAs in ovarian follicular development so far have mainly focused on either the physiology of antral follicular growth or on ovarian pathologies, such as PCOS. Information regarding the role of lncRNAs in granulosa cell apoptosis is limited and mainly involves in vitro studies. In the human granulosa-like tumor cell line KGN it is demonstrated that depending on the circumstances lncRNAs can be involved in either preventing or stimulating cellular apoptosis. For instance, lncRNA BANCR inhibits KGN cell proliferation and induces apoptosis [13], while the lncRNA HCP5 has the opposite effect in this cell line, promoting cell proliferation and inhibiting apoptosis via interaction with the miR-27a-3p/IGF-1 axis [14]. In line with these in vitro studies our RNA-seq and bioinformatic analysis data on porcine granulosa cells of AA and HA follicles show that apoptosis related pathways including “MAPK signaling pathway” “HIF-1 signaling pathway”, “TGF-beta signaling pathway”, “TNF signaling pathway”, “p53 pathway”, and “Apoptosis” involved in granulosa cells apoptosis and thus antral follicular atresia [15,16,17], come to the fore in AA follicles when performing pathway analysis for potentially targeted genes of DE-lncRNAs. Further strengthening to our hypothesis that lncRNAs are possibly involved in granulosa cells apoptosis comes from a correlation analysis based on Pearson coefficient. This analysis reveals that the up-regulated lncRNAs are significantly associated with expression of apoptosis related target mRNAs in granulosa cells from AA follicles, indicating a direct role of lncRNAs in antral follicular atresia.

Granulosa cell apoptosis is a tightly controlled process depending on the balance between anti-apoptotic and pro-apoptotic factors [18]. One of the key-antiapoptotic factors in granulosa cells of antral follicles is estradiol that is formed in these cells by the conversion of androgens by the enzyme aromatase (encoded by the CYP19A gene). Three aromatase gene isoforms, CYP19A1, 2, and 3 with >99% homology, are expressed in the porcine ovary [19]. We show here that CYP19A1 is a downstream potential mRNA target of NOVEL_00001850, the lncRNA that is highly downregulated in granulosa cells of AA follicles. This association between NOVEL_00001850 and reduced CYP19A1 gene expression is further confirmed by a lower aromatase protein expression in granulosa cells of AA follicles compared with HA follicles [20], and is presumably responsible for the previously observed lower estradiol follicular fluid concentrations in AA follicles [17]. We therefore hypothesize that NOVEL_00001850 might promote apoptosis of granulosa cells possibly by binding ssc-miR-125a, resulting in down-regulation of the CYP19A1 gene. The underlying molecular mechanism regarding how NOVEL_00001850 is involved in granulosa cell apoptosis needs to be further investigated. 

In our previous study using the same porcine ovary model we have shown that another novel type of ncRNAs, circRNAs, may also be involved in antral follicular atresia [17]. Through RNA-seq analysis, we identified 62 DE-circRNAs in granulosa cells between HA and AA follicles with characteristics of circRNAs such as back-splicing, and RNase R resistance and stability. Our results showed that one of the most upregulated DE-circRNAs, circ_KIF16B, may be involved in granulosa cells apoptosis through regulation of its targeted apoptotic gene *TP53* and its downstream target PHLDA3. These results point out that circRNAs, like lncRNAs, may be involved in granulosa cells apoptosis possibly by regulating the expression of their targeted protein-coding genes. To obtain a more complete overview of the genes involved in antral follicular atresia, we further analyzed the mRNA transcriptome profiles changes in porcine granulosa cells of advanced AA follicles [20]. Granulosa cell RNA-seq data revealed 2160 differentially expressed genes (DEGs), 1483 with higher and 677 with lower mRNA concentrations in AA follicles. Careful pathway analysis showed that the upregulated genes in AA follicles were highly enriched in inflammation and apoptosis processes, while the downregulated transcripts were mainly highlighted in the steroid biosynthesis pathway including genes involved in steroidogenesis (e.g., *CYP19A1, LHCGR*) and response to oxidative stress processes including antioxidant genes (e.g., *GSTA1*, *GCLC*, *GCLM, IDH1*, *GPX8*). Based on these observations, we hypothesize that aberrantly expressed circRNAs and lncRNA together take part in the induction and progression of granulosa cells apoptosis, and thus potentially in the transcriptional and posttranscriptional regulation of antral follicular atresia [5]. The detailed molecular pathways however are pending to be further investigated. 

It has long been assumed that FSH, LH, and estrogens are the main hormones that regulate porcine ovarian antral follicular development as well as the decision whether a small antral follicle is selected into the growing pool or remains dormant. The porcine estrous cycle spans a period of 18–24 days, which consists of a luteal phase and a follicular phase. Following luteolysis, a cohort of antral follicles is recruited from the small antral follicle pool and initiate. Within this cohort of recruited follicles several follicles grow faster than other follicles presumably due to enhanced FSH and/or LH receptor expression and these follicles produce increasing amounts of estradiol and inhibins, which have a negative feedback effect on the hypothalamus [1,21]. This results in a reduction in GnRH, LH and especially FSH release (due to the effects of inhibin) and subsequently estradiol and growth factor synthesis and release. As smaller antral follicles have insufficient LH receptors and are therefore more dependent on FSH, they will undergo degeneration when peripheral FSH levels decline [21]. Therefore, in the remainder of the follicular phase, following a further development of these LH-dependent larger sized follicles, an increased number of the smaller and medium-sized follicles will undergo atresia mainly due to granulosa cells apoptosis [21]. Given that our present study shows that lncRNAs are possibly involved in granulosa cells apoptosis and thus antral follicular atresia, we hypothesized these lncRNAs may contribute to follicle section during the follicular phase. Further research is necessary to determine the exact lncRNA-miRNA-RNA network and its target genes in granulosa cells and how this influences the process of apoptosis.

## 4. Materials and Methods

### 4.1. Animal and Follicle Collection

The granulosa cells used in this study were collected in a previous experiment [17]. In brief, 60 porcine ovaries from 30 gilts (nulliparous, around 180 days old with a bodyweight of approximately 120 kg) were used. Six ovaries from six different animals were snap-frozen immediately after slaughter in liquid nitrogen and stored at −80 °C until further immunohistochemical processing. The other 54 ovaries were immediately after slaughter washed in 0.01 M phosphate-buffered saline (PBS) pH 7.4, transferred to a 30 °C PBS solution containing 1% penicillin–streptomycin, and transported to the laboratory within 1 h after slaughter for follicle dissection. To ensure that follicle dissection was finished within a reasonably short time frame, the sows were slaughtered at three different days (three different batches). At each slaughter day, 15 to 20 ovaries were collected for antral follicle dissection. While dissecting one ovary, the remaining ovaries of that batch were stored in Dulbecco’s phosphate buffered saline (DPBS) on ice. For each batch, healthy antral follicles and atretic antral follicles 4–7 mm in diameter were dissected at random within 1.5 h after arrival in the lab. Antral follicle size was estimated by measurement of two perpendicular diameters with a millimeter scale.

Follicles were classified as healthy antral (HA) or atretic antral (AA) follicles based on the presence or absence of blood vessels in the follicle wall and the degree of follicular fluid clarity as determined by analysis under a stereomicroscope [22,23,24,25]. Briefly, HA follicles were characterized by a pinkish color, obvious vascular sheath on the follicular surface and clear follicular fluid in the antrum. AA follicles were identified by an opaque color, the absence of obvious vascularization on the surface of the follicle, and a large number of debris floating in the follicular fluid [22,25,26]. Using the criteria from Gioia et al. [26], these AA follicles were considered to be in an advanced stage of atresia. To further confirm the correctness of this morphological classification, histological analysis using HE staining and immunohistochemical staining with the apoptosis marker cleaved Caspase 3 were performed as reported previously [17]. 

Based on the criteria described above, 120 follicles (60 HA follicles and 60 advanced AA follicles) were selected from a pool of 235 follicles; the 115 follicles that were not included in the follicle selection did not meet our selection criteria for healthy and advanced atretic antral follicles and were thus excluded from sampling. These two pools of 60 follicles were randomly divided over six groups of 10 follicles each. Follicular fluid was collected and pooled per 10 follicles. In advanced stage AA follicles, apoptotic granulosa cells had largely become lose from the follicle wall and were floating in the antrum, mixing with the follicular fluid. When dissecting these follicles, the follicular fluid and floating apoptotic granulosa cells were collected together in a petri dish. The mural granulosa cells of these atretic follicles were scraped from the follicle wall and mixed with the follicular fluid to obtain a complete sample of granulosa cells (floating and mural cells). To create comparable granulosa cell samples, the mural granulosa cells scraped from the walls of healthy antral follicles were also mixed with the follicular fluid (for details see [17]).

### 4.2. RNA library Construction and Illumina Sequencing 

Total RNA from granulosa cells of three randomly selected samples of pooled granulosa cells from 10 HA and 10 AA follicles, respectively, was isolated using TRIzol (Life Technologies, Shanghai, China) for the purpose of RNA-sequencing (RNA-seq). 

High throughput transcriptome sequencing was carried out by Cloud-Seq Biotech (Shanghai, China). Briefly, total RNA extraction and rRNA depletion were performed using the Ribo-Zero rRNA Removal Kit (Illumina, Shanghai, China) according to the manufacturer’s instructions. RNA libraries were constructed from rRNA-depleted RNA using the TruSeq Stranded Total RNA Library Prep Kit (Illumina) according to the manufacturer’s instructions. Quality and quantity of the libraries were checked by the BioAnalyzer 2100 system (Agilent Technologies, Palo Alto, CA, USA). Ten pM libraries were denatured, captured on Illumina flow cells, amplified in situ and finally sequenced for 150 cycles using the Illumina HiSeq 4000 sequencer according to the manufacturer’s instructions. All Illumina sequencing data have been submitted to the Gene Expression Omnibus (GEO) under accession number (GSE136589).

### 4.3. Data Analysis

Data analysis was performed according to Veno et al. [27] with minor modifications. Briefly, paired-end reads were harvested from the Illumina HiSeq 4000 sequencer; quality control was performed by Q30 after 3’ adaptor-trimming and the removal of low-quality reads by Fastp software (v1.9.3). The high-quality trimmed reads were used to analyze circRNAs [17], mRNAs [20] and lncRNAs, respectively. 

For lncRNA data analysis, the high-quality reads were aligned to the pig reference genome (Sscrofa10.2) using Hisat2 software (v2.0.4) and porcine lncRNA database (http://lnc.rnanet.org/ accessed on 6 September 2020). Then, guided by the Ensembl gtf gene annotation file, Cuffdiff software was used to obtain the FPKM as expression profiles of lncRNA. Fold change and p-value were calculated based on FPKM and differentially expressed lncRNAs were identified. LncRNA potential target genes were predicted by their locations to nearby genes. GO and KEGG analysis were performed on these potential target genes using the Database for Annotation, Visualization and Integrated Discovery (DAVID) v6.8 [28]. The Correlation Matrix Heatmap was based on Pearson correlation coefficients that were calculated using the relevant FPKM values.

### 4.4. Quantitative Real-Time RT-PCR

Quantitative Real-Time RT-PCR (qRT-PCR) was used to validate the RNA-seq data, as described previously [29]. qPCR was carried out in an independent set of healthy and atretic follicles. One μg RNA of each sample was used for cDNA synthesis using the PrimeScript RT Master Mix (Takara, Guangzhou, China). Quantitative RT-PCR reactions were performed employing iQ SYBR Green Supermix (Bio-Rad, Guangzhou, China). Individual samples were measured in duplicate. A standard curve using serial dilutions of pooled sample (cDNA from all samples), a negative control without cDNA template, and a negative control without reverse transcriptase (RT) were included in every assay. Only standard curves with efficiency between 90% and 110% and a correlation coefficient above 0.99 were accepted. Data were normalized against the reference gene *Rps18*, which was chosen based on stable gene expression levels (geNorm; Ghent University Hospital, Ghent, Belgium). Primers were designed using the National Center for Biotechnology Information Primer-Blast (http://www.ncbi.nlm.nih.gov/ accessed on 6 July 2020). The primers used and PCR annealing temperatures for each gene are summarized in Table S1.

### 4.5. miRNA Target Prediction and lncRNA-miRNA-mRNA Network Construction

The predicted miRNA binding sites for the differentially expressed lncRNAs were identified using the miRanda [30] and Targetscan [31] algorithms. Only those lncRNA-miRNA interactions predicted by both algorithms were used for the downstream network construction and analyses. Next, mRNAs differentially expressed between HA and AA follicles were chosen as the candidates to be predicted as the targeted downstream genes of miRNAs. miRNA-mRNA interactions that were common in both miRanda and TargetScan were then used to determine the gene targets of each filtered miRNA. Using these data, the outline of a lncRNA-miRNA-mRNA regulatory network was visualized using Cytoscape (version 3.8). In addition, GO and KEGG pathway analyses was performed on these targeted mRNA transcripts.

### 4.6. Statistical Analysis

To perform the statistical analysis as described above, GraphPad Prism version 8.00 (Graphpad Software, San Diego, CA, USA) was used. Data were expressed as mean ± standard error of the mean (SEM). Data was checked for normality and when normality was confirmed the Student’s t test was used for data analysis. If normality could not be assumed, data were log10 transformed. If data was still not normally distributed after log transformation, a Mann–Whitney non-parametric test was used. *p* values <0.05 were considered to be significantly different.

## 5. Conclusions

Taken together, the results of the present study demonstrate for the first time the existence of differences in lncRNAs profiles in granulosa cells derived from HA and AA follicles. The DE-lncRNAs potential target genes appear to be involved in many molecular processes related to ovarian follicular development including apoptosis. The association between one of the most downregulated transcripts NOVEL_00001850 and its potentially targeted gene *CYP19A1* implies a potential role for lncRNAs in antral follicular atresia.

## Figures and Tables

**Table 1:** The top 20 upregulated and downregulated lncRNAs.

lncRNA	Fold Change	*p*_Value	FDR	Length
NOVEL_00009168	31.98	0.0028	0.0456	809
NOVEL_00008847	26.18	0.0046	0.0650	785
NOVEL_00007496	14.09	0.0001	0.0035	6036
NOVEL_00010018	9.81	0.0001	0.0019	967
ENSSSCT00000035867	9.74	0.0007	0.0165	7378
NOVEL_00010203	8.76	0.0207	0.1809	1304
NOVEL_00005276	7.77	0.0015	0.0286	1131
NOVEL_00008848	7.74	0.0001	0.0019	9018
NOVEL_00003234	6.75	0.0251	0.2047	1322
NOVEL_00005131	6.30	0.0170	0.1592	1048
NOVEL_00008054	6.07	0.0227	0.1904	893
NOVEL_00001799	5.90	0.0316	0.2343	1427
NOVEL_00006095	5.45	0.0259	0.2083	1018
NOVEL_00004604	5.17	0.0294	0.2242	1063
NOVEL_00009867	5.01	0.0410	0.2746	529
NOVEL_00008849	4.93	0.0256	0.2065	622
NOVEL_00005004	4.75	0.0001	0.0019	2409
NOVEL_00007680	4.69	0.0465	0.2969	1044
NOVEL_00008285	4.62	0.0391	0.2677	708
NOVEL_00006968	4.53	0.0366	0.2562	4021
NOVEL_00002285	−7.08	0.0137	0.1381	2041
NOVEL_00001850	−6.53	0.0027	0.0444	3650
NOVEL_00010591	−4.88	0.0372	0.2593	1013
NOVEL_00008412	−4.59	0.0011	0.0223	1030
NOVEL_00009434	−4.32	0.0168	0.1578	1485
NOVEL_00004206	−4.29	0.0486	0.3035	395
NOVEL_00005528	−3.68	0.0001	0.0019	853
NOVEL_00010234	−3.52	0.0213	0.1832	936
NOVEL_00003953	−3.52	0.0068	0.0873	4486
NOVEL_00008369	−3.25	0.0082	0.0996	451
NOVEL_00010618	−3.06	0.0373	0.2596	1941
NOVEL_00001195	−2.85	0.0071	0.0901	1024
NOVEL_00005529	−2.83	0.0156	0.1507	934
NOVEL_00004213	−2.64	0.0118	0.1254	1450
NOVEL_00001746	−2.47	0.0001	0.0019	5086
NOVEL_00010414	−2.42	0.0480	0.3013	3887
NOVEL_00009106	−2.36	0.0272	0.2152	1993
NOVEL_00004800	−2.32	0.0408	0.2736	672
NOVEL_00005624	−2.30	0.0172	0.1601	4900
NOVEL_00001230	−2.10	0.0352	0.2503	560

## Data Availability

All datasets generated for this study are included in the article Supplementary Materials.

## Supplementary Materials

The following are available online at https://www.mdpi.com/1422-0067/22/5/2677/s1, Table S1: Sequences of the primers used for qRT-PCR.
