# Supplementary material for: Characterization of Long Non-Coding RNA Profiles in Porcine Granulosa Cells of Healthy and Atretic Antral Follicles: Implications for a Potential Role in Apoptosis
Source: Int J Mol Sci. 2021 Mar 6;22(5):2677. doi: 10.3390/ijms22052677 (PMC7962063; doi:10.3390/ijms22052677)
Supplement: Supplementary file 1 [file ijms-22-02677-s001.pdf]

Table S1 Sequences of the primers used for qRT-PCR.

Circle Gene symbol		Primers (5'-3')	Anealing temp (°C)	Length of product
NOVEL_00001850	Forward	TCAGCTAGGCAATGCTGTGA	60.00	129
	Reverse	GAAGGACGAGATGTCAGGTGG		
NOVEL_00009434	Forward	GCTGCCTGGATTTCCCTGTAT	59.00	136
	Reverse	TGTTGCCCCATGTTTATGCAC		
NOVEL_00008412	Forward	TACGTGCATGCTCACCTCTT	60.00	117
	Reverse	GTGCGGACCGAGGTAATGTA		
NOVEL_00005529	Forward	ATCACCCAGGAGTGTCACAG	60.00	106
	Reverse	TTGGTACCAGTCAAGCAGGG		
NOVEL_00005528	Forward	TCTGCTTCCAACCAATCCCA	59.00	133
	Reverse	GTGCCTTCGGAGTTCCTCTT		
NOVEL_00006124	Forward	GCGTGAGGAATGGAAGGTCA	60.00	149
	Reverse	AAATCACTGCGGCAAAGCAG		
ENSSSCT00000018610	Forward	GGAGCGTCACTCCCCTCT	60.00	158
	Reverse	GTGGTGTCTGGAGACAGTGG		
NOVEL_00005276	Forward	TCAGATGGGATCGGGGGTAG	60.00	118
	Reverse	GCTGAAAAGCTCAGTGGCTG		
NOVEL_00009219	Forward	AAGAGCGGCCCGAGATCA	60.00	208
	Reverse	CCACCTGAGGGTCATCGTG		
NOVEL_00004669	Forward	TAAGTAAAAGCGGGGGAGCG	60.00	149
	Reverse	TCCACATCTGGTGCGAAGAC		
	Reverse	AGTGGTGTTTGCTTAGACGC		
RPS18*	Forward	ATTGCCTTTGCTATCACTGCG	60.00	132
	Reverse	GGTGATTACACGTTCCACCTCA		

Gene denoted with an asterisk was used as reference gene for normalization.
